# Obituary Notice of Professor Power

**Published:** 1853-04

**Authors:** 


### OBITUARY NOTICE OF PROFESSOR POWER.

We had the pleasure of a slight personal acquaintance with the gifted Professor of Theory and Practice of Medicine, in the University of Maryland, the late William Power, M. D. The impression which he made upon us in a single, brief interview was exceedingly agreeable. We were pained to hear of his early death, which took place in Baltimore, on the 15th of last August. A beautiful tribute to his memory appeared in the last number of the American Journal of the Medical Sciences, from the pen of his fellow-student and friend, Dr. Alfred Stillé, of Philadelphia. He was an uncommon man, and, if he had lived, would unquestionably have risen to great distinction. From the eloquent and discriminating obituary notice of Dr. Stillé, we make a few extracts which, we are sure, will be read with interest.

"The career of Dr. Power as professor was a distinguished one. That it was singularly successful is proved by the large number of students whom he drew to the University; by the position he at once assumed among his professional brethren, and by the reputation he soon acquired as a clinical teacher in the College Infirmary. His thorough, systematic, and intelligent study of disease, his accurate diagnosis, and his mode of illustrating one case by another, interested his audience deeply, and accustomed them to observe for themselves. In a word, his whole system of analytical study, as he had learned it from the ablest teachers of Europe, attracted and delighted all of the maturer and best-furnished minds among his hearers, and gradually raised up a class of ardent cultivators of medical science, which has already given to the world some of the fruits of his instruction, and some earnest of their own future attainments. As a lecturer, we are informed, he did not aim at originality, but rested satisfied if what he taught, from whatever source obtained, was *true*. The confidence he inspired was boundless; for did he make a wrong diagnosis, or misstate a fact, he was the first to admit it when discovered, and to confess his error. His lectures were less remarkable for excellence of style and originality of thought, than for their presenting a calm and unbiassed arrangement of the opinions of others, corrected and modified by his own experience. In this, as in all else, he held truth to be more valuable than ingenuity, and a positive result than the most elaborate and cunningly devised fiction. Yet, in dealing with the errors and follies of medical systems, he was careful not to forget the charity which is due to the weakness of human nature, nor the distinction, too often lost sight of, between the doctrines he combated and the persons who propounded or adopted them.

"Dr. Power's health had never been robust. Emphysema of the lungs, and a consequent derangement of the heart's action, had, for a long time, made active exertion fatiguing to him. As long ago as 1837, a pedestrian tour in Switzerland cost him several paroxysms of severe dyspnoea and palpitation of the heart with hæmoptysis; but he did not become seriously alarmed about his situation until nearly six years afterwards. In the autumn of 1843, he was obliged to seek relaxation in Cuba, whence he returned in the following spring, with his health materially improved. He continued better, in the main, although far from being vigorous, and from time to time he showed the effects of too close an application to his duties as hospital physician. His marriage in 1847, and his appointment in the University, which he had received two years before, exerted, doubtless, a very favorable influence upon his health, for he continued to perform with animation and a good degree of vigor his professorial duties, to draw around him a crowd of earnest and attached pupils, and in the social circle to win by his amenity, and interest by his varied talents, a large number of sincere and valuable friends. But the disease which had so long been baffled by skill and sedulous attention at length gave signs that it was obtaining the mastery. During the winter of 1851–52, Dr. P. was unable to perform his public duties, and, in February of the latter year, resigned his professorship. In the month of May, the writer of this notice had the satisfaction of an interview with his old companion. He was wasted and wan indeed, for consumption had nearly worn away his earthly dwelling; but his soul shone all the clearer, and brighter, and purer for its freedom from the clogs of flesh. One might well have expected to hear the devotee of science, and the ambitious teacher, mourn over the unfinished career he had run, and resent the defeat of his wishes. But his spirit had been chastened. Contented with his share of worldly success, without a syllable of complaint at the disappointment of his hopes, he could still take interest in the science which he loved, and find in conversation upon its progress a consolation for his own withdrawal from a share in its rewards. Resigned and cheerful, he awaited with patience, yet with strong desire, the moment of his departure; for, while he longed for a release from suffering, he did not disdain the world in which he had been permitted to fit himself for a better.

"For three months after this interview he waxed feebler and feebler, but his soul was more and more brightened by a light from beyond the grave, and displayed to the last moment the calm hopefulness of one whose heart is fixed on Heaven."

